# Voronoi cell finite element method for heat conduction analysis of composite materials

**DOI:** 10.1038/s41598-024-61263-4

**Published:** 2024-05-27

**Authors:** Siqi Chen, Changhao Hu, Jingjie Tian, Dawen Tan, Yuqiang Gong, Fan Xia, Shaoqing Ning, Rui Zhang

**Affiliations:** 1 Faculty of Civil Engineering and Mechanics, Kunming University of Science and Technology, Kunming, 650500 China; 2 Xiluodu Hydropower Plant, China Yangtze Power Co., Ltd., Zhaotong, 657000 China

**Keywords:** Voronoi cell finite element, Effective thermal conductivity, Heat flux, Temperature, Composite material, Engineering, Civil engineering, Mechanical engineering, Composites

## Abstract

In this paper, Voronoi cell finite element method (VCFEM) based on assumed flux hybrid formulation has been presented for heat conduction problem of particle reinforced composites material. The heat fluxes satisfying a priori internal thermal balance are directly approximated independently in the matrix and the inclusion respectively. The temperatures on element boundary and matrix-inclusion interface are interpolated by nodal temperature. The thermal balance on the interelement boundary and matrix-inclusion interface is relaxed and introduced into the functional by taking the temperature as Lagrange multiplier. In this way, a functional containing two variables of heat flux and temperature is proposed. Full field heat flux and effective thermal conductivity are obtained. Feasibility and effectiveness of the proposed approach are verified through several numerical examples.

## Introduction

Particle reinforced composites are widely used in engineering to improve the performance of the matrix material by adding reinforcements to the matrix material. In particle reinforced composites, when a uniform steady heat flux is disturbed by the presence of inclusions in the process of heat transfer, there is a local intensification of temperature gradients, which will thus cause great thermal stress and other adverse consequences, further affecting the reliability of structural components^1,2^.

The study of the behavior of the microscopic heat flux distribution is of great practical importance. Also, the effective heat conductivity is an important parameter for the macroscopic problem. Numerical methods are usually adopted to solve the heat conduction problems of heterogeneous materials. Finite element method (FEM) is a sophisticated calculation tool and can be applied to heat conduction problems. The calculation accuracy of finite element method depends on the size of grids. For complex geometric shapes, a large number of fine meshes are required. Therefore, when calculating the performance of heterogeneous materials, it is necessary to divide a large number of fine grids at the interface between inclusions and matrix, so that the calculation area is limited in a very small area. To overcome these difficulties, Moorthy et al.^3^ proposed the Voronoi cell finite element method (VCFEM) based on the stress hybrid element^4^. Voronoi’s grid can reflect the randomness of the size, shape and spatial distribution of inclusions and can analyze the model containing a large number of inclusions, presenting a wide applicability. This method has less mesh generation and high calculation accuracy. Since this method was put forward, various VCFEM have been established according to the different heterogeneities of the internal microstructure of composite materials. VCFEM first proposed for heterogeneous composite materials is divided into two group, one is transformation strain method and the other is direct implementation of interface constraint. In the transformation strain formulation, strain fields are discontinuous while stress fields are continuous. In the latter formulation, both strain fields and stress fields are discontinuous. The latter method has proven to be more effective for stress analysis in the literature^3^ and has been developed in all subsequent studies for stress analysis. Special VCFEM for voids^5,6^ , inclusions^7,8^ and cracks^9,10^ are established and interior stress field are calculated and corresponding post-processing calculations have been carried out. These special VCFEMs are based on assumed stress hybrid formulation. Both stress and the displacement are independently assumed in the inertia and on the edge of element respectively. The stress field is calculated directly in VCFEM, and it does not need to be obtained by displacement derivative as in finite element method. Therefore, the precision of the results thus obtained from VCFEM is high.

VCFEM^11^ based on hybrid flux model and transformation temperature gradient method for materials imbedded with holes were proposed for steady-state heat conduction problem. The variational theory used in the heat conduction process is similar to the transformation strain method in the elastic theory which has proved to be less effective compared with direct constraint method. Special Voronoi elements^12^ based on fundamental solutions are built for analyzing clustering on thermal effect in fiber reinforced cement composites. Fundamental solutions need to be constructed for special example, it does not have universal applicability for arbitrary shapes and fibers shape in that paper are all circler.

Considering advantages of VCFEM in calculating particle reinforced composites, a new VCFEM based on hybrid flux model and direct constraint method is proposed for heat conduction problem. In this model, the heat flux in the domain of each element and the temperature on the element boundary are assumed independently. The heat flux in the matrix and the inclusion satisfying thermal balance are assumed independently. Thermal balance on interelement boundary and matrix-inclusion interface inside the element are relaxed by temperature as Lagrange multiplier introduced into the functional. Thus, the functional containing two variables of heat flux and temperature is built (“Functional derivation”). Heat flux approximations satisfying equilibrium relations in matrix and inclusion are built up from the heat flux function respectively (“Equilibrated flux fields in VCFEM”). Temperatures on element boundary and matrix-inclusion interface are interpolated by nodal temperature. The heat flux parameters inside the element can be linked with the displacements of the internal node and the external node of the element, and can be condensed together with the displacements of the internal node, thus obtaining the same standard solution form as the traditional FEM (“Method of solution”). Numerical examples are shown in “Numerical examples” and conclusions are drawn in “Conclusions”.

## The Voronoi cell FEM (VCFEM) formulation

### Functional derivation

**Figure 1:**
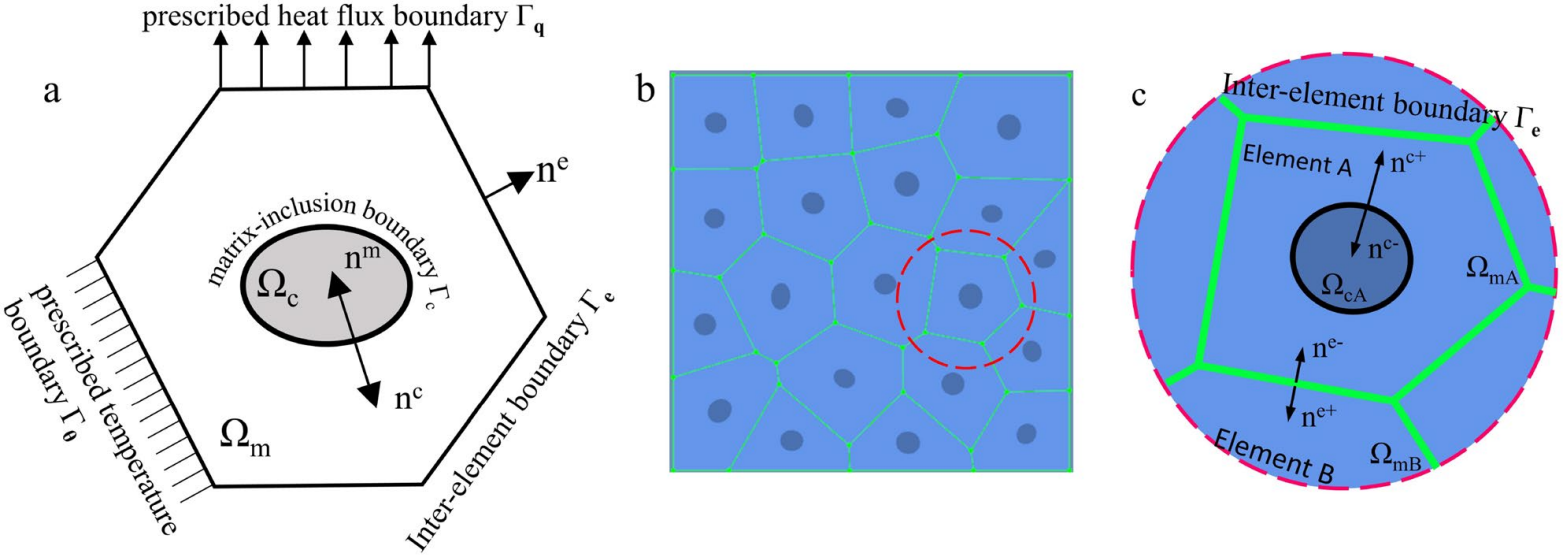
(**a**) The typical Voronoi element. (**b**) Voronoi mesh for materials with various inclusions. (**c**) The schematic diagram of each outer normal of ‘element A’ as the research object.

Microstructure containing inclusions is tessellated by Voronoi mesh, with each element containing an inclusion as shown in Fig. 1b. The matrix region of each element is Ω_m_, the inclusion region is Ω_c_. In the Fig. 1a it is seen that the element boundary Γ consists of inter-element boundary Γ_e_, prescribed temperature boundary Γ_θ_ and prescribed heat flux boundary Γ_q_ with outward normal n^e^(Γ = Γ_e_ ∪ Γ_θ_ ∪ Γ_q_). Γ_c_ is matrix-inclusion interface with an outward normal $n^{{\text{c}}} = \left\{ {{\text{n}}_{{\text{x}}}^{{\text{c}}} {\text{,n}}_{{\text{y}}}^{{\text{c}}} } \right\}$. The heat flux vector ${\text{q}}^{m}$ with cartesian component (${\text{q}}_{\rm{x}}^{{\text{m}}}$,${\text{q}}_{\rm{y}}^{{\text{m}}}$) in the Ω_m_ and the heat flux vector ${\text{q}}^{{\text{c}}}$ with cartesian component (${\text{q}}_{\rm{x}}^{{\text{c}}}$,${\text{q}}_{\rm{y}}^{{\text{c}}}$) in the Ω_c_ are assumed based on satisfying the self-equilibrating heat flux as shown in Eq. (1) (excluding external heat transfer). This will be explained and justified in “Equilibrated flux fields in VCFEM”.

1 $$\begin{gathered} \frac{{\partial {\text{q}}_{\rm{x}}^{{\text{m}}} }}{{\partial \rm{x}}} + \frac{{\partial {\text{q}}_{\rm{y}}^{{\text{m}}} }}{{\partial \rm{y}}} = 0{\text{ in }}\Omega_{m} \hfill \\ \frac{{\partial {\text{q}}_{\rm{x}}^{{\text{c}}} }}{{\partial \rm{x}}} + \frac{{\partial {\text{q}}_{\rm{y}}^{{\text{c}}} }}{{\partial \rm{y}}} = 0{\text{ in }}\Omega_{c} \hfill \\ \end{gathered}$$

And Fourier’s law for the constitutive relation of heat flux is:

2 $$\begin{gathered} {\mathbf{q}}^{m} = - {\varvec{k}}_{m} \nabla \rm{\theta }^{m} {\text{ in }}\Omega_{m} \hfill \\ {\mathbf{q}}^{c} = - {\varvec{k}}_{c} \nabla \rm{\theta }^{c} {\text{ in }}\Omega_{c} \hfill \\ \end{gathered}$$

where k_m_ and k_c_ are the thermal conductivity matrices of the matrix and inclusion, respectively. *θ*^m^ and *θ*^c^ are the corresponding temperatures.

with the boundary conditions

3 $$\theta^{e} { = }\overline{\theta } {\text{on}} \Gamma_{\theta }$$

4 $$n^{{\text{e}}} q^{m} = {\overline{\text{q}}}_{n} {\text{ on }}\Gamma_{q}$$

where θ^e^, $\overline{\theta }$ and ${\overline{\text{q}}}_{n}$ are the displacement of the element boundary in Eq. (9), the prescribed temperature on Γ_θ_ and the prescribed heat flux on Γ_q_, respectively.

The outward normal heat flux ${\text{q}}_{{\text{n}}}^{{\text{m}}}$ of the element and the heat flux ${\text{q}}_{{\text{n}}}^{{\text{c}}}$ in the outward normal direction at the matrix-inclusion interface can be expressed as

5 $$\begin{gathered} {\text{q}}_{{\text{n}}}^{{\text{m}}} { = }{\text{n}}^{e} {\text{q}}^{{\text{m}}} {\text{ on }}\Gamma_{{\text{e}}} \hfill \\ {\text{q}}_{{\text{n}}}^{{\text{m}}} = {\text{n}}^{{\text{m}}} {\text{q}}^{{\text{m}}} {\text{ on }}\Gamma_{{\text{c}}} \hfill \\ {\text{q}}_{{\text{n}}}^{{\text{c}}} { = }{\text{n}}^{{\text{c}}} {\text{q}}^{{\text{c}}} {\text{ on }}\Gamma_{{\text{c}}} \hfill \\ \end{gathered}$$

As shown in Fig. 1c, the outer normals **n**^e^, **n**^m^ and **n**^c^ at the inter-element boundary and matrix-inclusion boundary have the following transformation relations:

6 $$\begin{gathered} {\text{n}}^{e} { = }{\text{n}}^{e + } {\text{ on }}\Gamma_{{\text{e}}} \hfill \\ {\text{n}}^{{\text{c}}} { = }{\text{n}}^{{\text{c + }}} {, }{\text{n}}^{m} { = }{\text{n}}^{{\text{c - }}} {\text{ on }}\Gamma_{{\text{c}}} \hfill \\ \end{gathered}$$

where the superscripted plus and minus signs are just to visually show the orientation of the normals of neighboring regions on the common boundary.

Further, the following conditions of heat flux continuity are also satisfied on the matrix-inclution boundary Γ_c_ and the inter-element boundary Γ_e_.

7 $$\begin{gathered} n^{{{\text{c}} - }} q^{{\text{m}}} { + }n^{{\text{c + }}} q^{{\text{c}}} {\text{ = 0 on }}\Gamma_{{\text{c}}} \hfill \\ n^{{\text{e + }}} q^{ + } { + }n^{{{\text{e}} - }} q^{ - } = 0{\text{ on }}\Gamma_{{\text{e}}} \hfill \\ \end{gathered}$$

where **q**^+^ and **q**^-^ denote the heat fluxes of neighboring cells on the inter-cell boundary, respectively.

The energy functional $\Pi_{e}$ may be expressed for each element in terms of heat flux and boundary/interface temperature fields as

8 $$\begin{gathered} {\Pi _e} = \frac{1}{2}\int_{{\Omega _m}} {{q^{\text{m}}}^T{{\text{S}}_{\text{m}}}{q^{\text{m}}}} {\text{d}}\Omega {\text{ + }}\frac{1}{2}\int_{{\Omega _c}} {{q^{\text{c}}}^T{{\text{S}}_{\text{c}}}{q^{\text{c}}}} {\text{d}}\Omega \\ + \int_{{{{\Gamma }}_e}} {\left( {{{\bf n} ^{e + }}{\bf q} _{}^{{ + }} + {{\bf n} ^{e - }}{\bf q} _{}^{{ - }}} \right){\theta ^{\text{e}}}} {{{\rm d}\Gamma }} + \int_{{{{\Gamma }}_\theta }} {{{\bf n} ^e}{\bf q} _{}^{\text{m}}\bar \theta } {{ {\rm d}\Gamma }} + \int_{{{{\Gamma }}_{\text{q}}}} {{\text{(}}{{\bf n} ^e}{\bf q} _{}^{\text{m}} - {{{{\bar {\rm q}}}}_{\text{n}}}){\theta ^{\text{e}}}{{{\rm d}\Gamma }}} \\ + \int_{{{{\Gamma }}_c}} {\left( {{{\bf n} ^{c - }}{\bf q} _{}^m + {{\bf n} ^{c + }}{\bf q} _{}^c} \right){\theta ^{\text{c}}}{{{\rm d}\Gamma }}} \\ \end{gathered}$$

Equation (8) can also be expressed as:

9 $$\Pi_{e} = \frac{1}{2}\int_{{\Omega_{m} }} {q^{{{\text{m}}T}} {\text{S}}_{{\text{m}}} q^{{\text{m}}} } {\text{d}}\Omega { + }\frac{1}{2}\int_{{\Omega_{c} }} {q^{{{\text{c}}T}} {\text{S}}_{{\text{c}}} q^{{\text{c}}} } {\text{d}}\Omega + \int_{{\Gamma }} {{\text{n}}^{e} {\text{q}}^{m} \theta^{{\text{e}}} } {\text{d}}\Gamma - \int_{{{\Gamma }_{{\text{q}}} }} {{\overline{\text{q}}}_{{\text{n}}} \theta^{{\text{e}}} {\text{d}}\Gamma } + \int_{{{\Gamma }_{c} }} {\left( {{\text{n}}^{c - } {\text{q}}^{m} + {\text{n}}^{c + } {\text{q}}^{c} } \right)\theta^{{\text{c}}} {\text{d}}\Gamma }$$

where Γ = Γ_e_ ∪ Γ_θ_ ∪ Γ_q_, and the element boundary temperature θ^e^ is equal to $\overline{\theta }$ when located at the prescribed temperature boundaries Γ_θ_.

The total energy $\Pi$ for the entire domain is obtained by adding each element functional $\Pi _{e}$ as:

10 $$\Pi { = }\sum\limits_{n = 1}^{e} {\Pi_{e} }$$

${\text{S}}_{{\text{m}}}$ and ${\text{S}}_{{\text{c}}}$ are the inverse of isotropic thermal conductivity matrix that are expressed as:

11 $${\text{S}}_{{\text{m}}} { = }\left[ {\begin{array}{*{20}c} {k_{{\text{m}}} } & 0 \\ 0 & {k_{{\text{m}}} } \\ \end{array} } \right]^{{{ - }1}} ,{\text{ S}}_{{\text{c}}} { = }\left[ {\begin{array}{*{20}c} {k_{{\text{c}}} } & 0 \\ 0 & {k_{{\text{c}}} } \\ \end{array} } \right]^{{{ - }1}}$$

Compatible temperature fields are continuous on the inter-element boundary and the matrix-inclusion interface. The temperature θ^e^ at the element boundary and the temperature θ^c^ at the matrix-inclusion interface are obtained by interpolating the nodal temperatures of the following form:

12 $$\theta^{{\text{e}}} { = }L\Theta^{{\text{e}}} {\text{ on }}\Gamma {\text{ and }}\theta^{{\text{c}}} { = }L\Theta^{{\text{c}}} {\text{ on }}\Gamma_{{\text{c}}}$$

where $L$ is the interpolating function, $\Theta^{{\text{e}}}$ and $\Theta^{{\text{c}}}$ are temperature at node of element boundary and matrix-inclusion boundary respectively.

### Equilibrated flux fields in VCFEM

**Figure 2:**
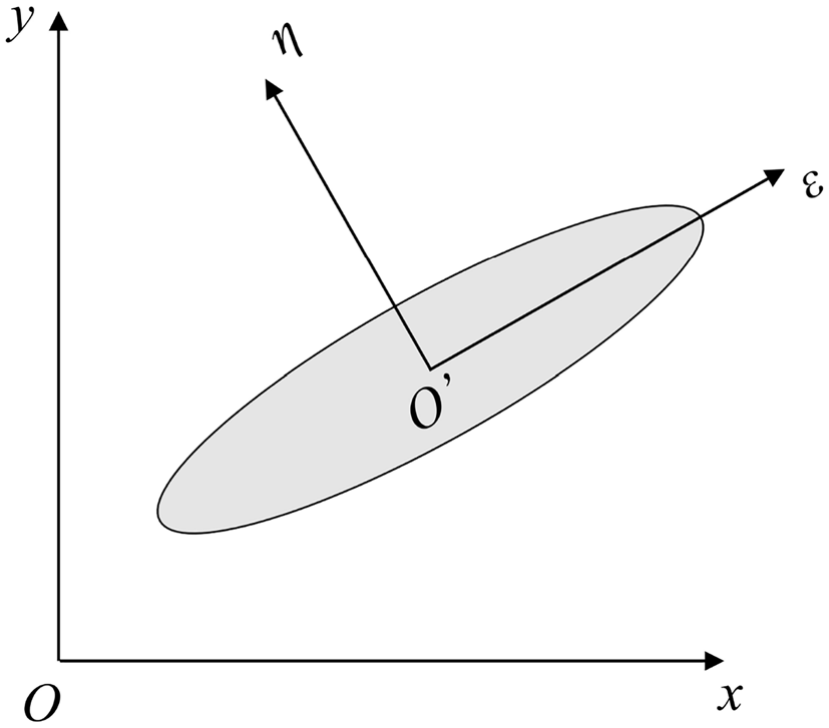
Local coordinate system for Voronoi cells.

In VCFEM formulation, the equilibrium conditions Eq. (1) in the matrix and inclusion respectively are satisfied a priori in a strong sense. In the absence of heat source, heat flux fields satisfying equilibrium relations can be built up from the heat flux function $\Phi$. In the local coordinate system, the resulting heat flux are

13 $$q_{x} = \frac{\partial \Phi }{{\partial \eta }},\begin{array}{*{20}c} {} & {q_{y} = - \frac{\partial \Phi }{{\partial \varepsilon }}} \\ \end{array}$$

where *ε* and* η* are localized coordinate as in Fig. 2.

Since bringing Eq. (13) into Eq. (1) yields a constant relationship, we assume that the heat flux field assumed in the form of Eq. (13) is presupposed to satisfy the equilibrium conditions.

Different functional forms of the heat flux functions are chosen for the matrix and inclusion phases in VCFEM implementation. Independent construction of heat flux functions $\Phi^{m}$ and $\Phi^{{\text{c}}}$ in matrix and inclusion allow for flux discontinuities across the matrix-inclusion interface.

The proper selection of heat flux function has a significant influence on the convergence and efficiency of VCFEM. The construction of heat flux functions should consider the influence of shape location and distribution of inclusion. The influence on the shape of matrix heat flux functions far from the interface should disappear. Heat flux function for matrix is constructed of pure polynomial forms $\Phi_{poly}^{m}$ and reciprocal functions^13^ $\Phi_{rec}^{m}$, and heat flux function for inclusion is constructed of pure polynomial forms $\Phi_{poly}^{c}$.The heat flux functions for the matrix and inclusion are constructed as:

14 $$\begin{gathered} \Phi_{{}}^{m} = \Phi_{poly}^{m} + \Phi_{rec}^{m} \hfill \\ \Phi_{{}}^{c} = \Phi_{poly}^{c} \hfill \\ \end{gathered}$$

The local coordinate system takes the center of the inclusion as the origin, and the horizontal and longitudinal axes correspond to the long half axis and the short half axis of the inclusion respectively. In the above function, the pure polynomial part is constructed from the Pascal triangle in the local coordinates (*ε* ,* η*) shown in Fig. 2, which is given by the following formula:

15 $$\Phi_{{{\text{poly}}}}^{{\text{m/c}}} = \sum\limits_{p = 0,q = 0}^{{p_{n} ,q_{n} }} {\varepsilon^{p} \eta^{q} \beta_{pq}^{{}} }$$

The term of the reciprocal function is expressed as:

16 $$\Phi_{rec}^{m} = \sum\limits_{p,q} {\xi^{p} \eta^{q} \left( {\frac{{\Delta \beta_{pq1} }}{{f^{p + q} }} + \frac{{\Delta \beta_{pq2} }}{{f^{p + q + 1} }} + \cdots } \right)}$$

The function $f$ has the following property when $(\varepsilon ,\eta ) \to 0$, 1/*f* → 0 , and can be used as a mapping function to construct a shape-based reciprocal heat flux function. Furthermore, at the matrix-inclusion interface, *f* = 1, and the coefficient $\Delta \beta$ is equal to the polynomial heat flux coefficient, ensuring that heat flux is continuous at the interface. In the region away from the inclusions, the effect of the interaction heat flux function can be overlooked. Thus, near the matrix-inclusion interface, the heat flux is a combination of polynomial and interaction components, and the closer to the interface, the stronger the effect, ensuring that the approach can better compute the heat flux concentration at the interface. According to this characteristic, it can be taken as an elliptic function.

The associative Eqs. (13)–(16), the heat flow density can be expressed as:

17 $$\left\{ {\begin{array}{*{20}c} {q_{x}^{m} } \\ {q_{y}^{m} } \\ \end{array} } \right\} = \left\{ {\begin{array}{*{20}c} {\sum\limits_{p,q}^{{p_{n} ,q_{n} }} {\frac{{\partial \left( {\varepsilon^{p} \eta^{q} } \right)}}{\partial \eta }\beta_{pq} + \sum\limits_{p,q}^{{p_{n} ,q_{n} }} {\frac{{\partial \left( {\varepsilon^{p} \eta^{q} f^{ - p - q} } \right)}}{\partial \eta }\Delta \beta_{pq} } } } \\ { - \sum\limits_{p,q}^{{p_{n} ,q_{n} }} {\frac{{\partial \left( {\varepsilon^{p} \eta^{q} } \right)}}{\partial \eta }\beta_{pq} - \sum\limits_{p,q}^{{p_{n} ,q_{n} }} {\frac{{\partial \left( {\varepsilon^{p} \eta^{q} f^{ - p - q} } \right)}}{\partial \eta }\Delta \beta_{pq} } } } \\ \end{array} } \right\} = \left[ {P_{poly} } \right]\left\{ {\begin{array}{*{20}c} {\beta_{11} } \\ \vdots \\ {\beta_{pq} } \\ \vdots \\ \end{array} } \right\} + \left[ {P_{rec} } \right]\left\{ {\begin{array}{*{20}c} {\Delta \beta_{11} } \\ \vdots \\ {\Delta \beta_{pq} } \\ \vdots \\ \end{array} } \right\}$$

18 $$\left\{ {\begin{array}{*{20}c} {q_{x}^{c} } \\ {q_{y}^{c} } \\ \end{array} } \right\} = \left\{ {\begin{array}{*{20}c} {\sum\limits_{p,q}^{{p_{n} ,q_{n} }} {\frac{{\partial \left( {\varepsilon^{p} \eta^{q} } \right)}}{\partial \eta }\beta_{pq} } } \\ { - \sum\limits_{p,q}^{{p_{n} ,q_{n} }} {\frac{{\partial \left( {\varepsilon^{p} \eta^{q} } \right)}}{\partial \eta }\beta_{pq} } } \\ \end{array} } \right\} = \left[ {P_{poly} } \right]\left\{ {\begin{array}{*{20}c} {\beta_{11} } \\ \vdots \\ {\beta_{pq} } \\ \vdots \\ \end{array} } \right\}$$

Therefore, each indeterminate field is expressed as product of the function** P** of localized coordinate alone and the heat flux coefficients **β** alone:

19 $$q^{m} = \left| {\begin{array}{*{20}c} {P_{poly}^{m} } & {P_{rec}^{m} } \\ \end{array} } \right|\left| {\begin{array}{*{20}c} {\beta_{poly}^{m} } \\ {\beta_{rec}^{m} } \\ \end{array} } \right| = P^{m} \cdot \beta^{m} \, , \, q^{c} = P^{c} \cdot \beta^{c}$$

Take Eqs. (12) and (19) into Eq. (9):

20 $$\Pi_{e} = \frac{1}{2}\beta^{mT} H^{m} \beta^{m} + \frac{1}{2}\beta^{c} H^{c} \beta^{c} + \beta^{mT} G_{e} \Theta^{e} + \beta^{mT} G_{m} \Theta^{c} + \beta^{cT} G_{c} \Theta^{c} - \overline{q}^{T} \Theta^{e}$$

where the **H**-matrix and **G**-matrix are defined as follows:

.

21 $$\begin{gathered} H^{{\text{m}}} { = }\int_{{\Omega_{{\text{m}}} }} {P^{{{\text{mT}}}} {\text{S}}_{{\text{m}}} P^{{\text{m}}} } {\text{d}}\Omega, \,\, H^{{\text{c}}} { = }\int_{{\Omega_{{\text{c}}} }} {P^{{{\text{cT}}}} {\text{S}}_{{\text{c}}} P^{{\text{c}}} } {\text{d}}\Omega \\ G_{e} = \int_{\Gamma } {P^{mT} } n^{eT} Ld\Gamma , \, G_{m} = \int_{{\Gamma_{c} }} {P^{mT} } n^{cT} Ld\Gamma\\ G_{{\text{c}}} { = }\int_{{\Gamma_{{\text{c}}} }} {P^{{{\text{cT}}}} } n^{{{\text{cT}}}} L{\text{d}}\Gamma, \,\, \overline{q}^{{\text{T}}} { = }\int_{{\Gamma_{{\text{q}}} }} {\overline{q}_{{\text{n}}}^{{\text{T}}} {\mathbf{L}}{\text{d}}\Gamma }. \end{gathered}$$

Where Γ = Γ_e_ ∪ Γ_θ_ ∪ Γ_q_, and the element boundary temperature θ^e^ is equal to $\overline{\theta }$ on the prescribed temperature boundaries Γ_θ_. ‘m’, and ‘c’ are superscripts used to distinguish the matrix and inclusion parts, respectively.

### Method of solution

Setting the primary variations of $\Pi_{e}$ in Eq. (20) with respect to the heat flux coefficients ${{\varvec{\upbeta}}}^{m}$ and ${{\varvec{\upbeta}}}^{{\text{c}}}$, respectively, to zero:

22 $$\frac{{\partial \Pi_{{\text{e}}} }}{{\partial \beta^{m} }}{ = }0,\frac{{\partial \Pi_{{\text{e}}} }}{{\partial \beta^{c} }}{ = }0$$

yields

23 $$\left[ {\begin{array}{*{20}c} {c^{m} } & 0 \\ 0 & {{\text{H}}^{c} } \\ \end{array} } \right]\left\{ {\begin{array}{*{20}c} {c^{m} } \\ {\beta^{c} } \\ \end{array} } \right\} = \left[ {\begin{array}{*{20}c} { - {\text{G}}_{e} } & {{\text{G}}_{m} } \\ 0 & { - {\text{G}}_{c} } \\ \end{array} } \right]\left\{ {\begin{array}{*{20}c} {\Theta^{e} } \\ {\Theta^{c} } \\ \end{array} } \right\} \Rightarrow {\text{H}} \beta = {\text{G}} \Theta$$

Heat flux coefficients ${{\varvec{\upbeta}}}$ from Eq. (23) can be written as

24 $$\beta = {\text{H}}^{ - 1} {\text{G}} \Theta$$

Setting the primary variations of the total energy functional $\Pi$ in Eq. (10) with respect to $\Theta^{{\text{e}}}$ and $\Theta^{{\text{c}}}$ to zero:

25 $$\frac{\partial \Pi }{{\partial \Theta^{e} }}{ = }0,\frac{\partial \Pi }{{\partial \Theta^{c} }}{ = }0$$

gives

26 $$\sum\limits_{e = 1}^{n} {\left[ {\begin{array}{*{20}c} { - {\text{G}}_{e} } & 0 \\ {{\text{G}}_{m} } & { - {\text{G}}_{c} } \\ \end{array} } \right]\left\{ {\begin{array}{*{20}c} {\beta^{m} } \\ {\beta^{c} } \\ \end{array} } \right\} = \sum\limits_{e = 1}^{n} {\left\{ {\begin{array}{*{20}c} { - \overline{q}} \\ 0 \\ \end{array} } \right\}} }$$

Take Eq. (24) into Eq. (26)

27 $$\sum\limits_{e = 1}^{n} {\left[ {\begin{array}{*{20}c} { - {\text{G}}_{e}^{T} {\text{H}}^{ - m} {\text{G}}_{e} } & {{\text{G}}_{e}^{T} {\text{H}}^{ - m} {\text{G}}_{m}^{{}} } \\ {{\text{G}}_{m}^{T} {\text{H}}^{ - m} {\text{G}}_{e} } & { - {\text{G}}_{m}^{T} {\text{H}}^{{{ - }m}} {\text{G}}_{m} - {\text{G}}_{c}^{T} {\text{H}}^{ - c} {\text{G}}_{c} } \\ \end{array} } \right]\left\{ {\begin{array}{*{20}c} {\Theta^{e} } \\ {\Theta^{c} } \\ \end{array} } \right\} = \sum\limits_{e = 1}^{n} {\left\{ {\begin{array}{*{20}c} {\overline{q}} \\ 0 \\ \end{array} } \right\}} }$$

28 $$\sum\limits_{{\text{e}}} {\left[ {\begin{array}{*{20}c} {K_{11} } & {K_{12} } \\ {K_{12}^{T} } & {K_{22} } \\ \end{array} } \right]\left[ {\begin{array}{*{20}c} {\Theta^{{\text{e}}} } \\ {\Theta^{{\text{c}}} } \\ \end{array} } \right]} = \sum\limits_{e} {\left[ {\begin{array}{*{20}c} {\overline{q}} \\ {\mathbf{0}} \\ \end{array} } \right]}$$

We may divide Eq. (28) into two parts. The first part relates external node temperature of the element with those of other elements, satisfying:

29 $$\sum\limits_{e} {(K_{11} \Theta^{{\text{e}}} + K_{12} \Theta^{{\text{c}}} )} = \sum\limits_{e} {\overline{q}}$$

The second part relates only within each element, since the internal node temperature of each element is only related to the external node temperature of itself. The relationship between them is satisfied as follows:

30 $${\mathbf{K}}_{21} \cdot \Theta^{e} + {\mathbf{K}}_{22} \cdot \Theta^{{\text{c}}} = 0$$

31 $$\Theta^{{\text{c}}} = - K_{22}^{ - 1} K_{12}^{T} \Theta^{{\text{e}}}$$

Substituting Eq. (31) into Eq. (29), the solved relation can be expressed as:

32 $$\sum\limits_{e} {{\text{K}}_{{\text{e}}} \Theta^{{\text{e}}} } = \sum\limits_{e} {\overline{q}}$$

where, ${\text{K}}_{{\text{e}}} { = }K_{11} - K_{12} K_{22}^{ - 1} K_{12}$ is the element stiffness matrix.

**Figure 3:**
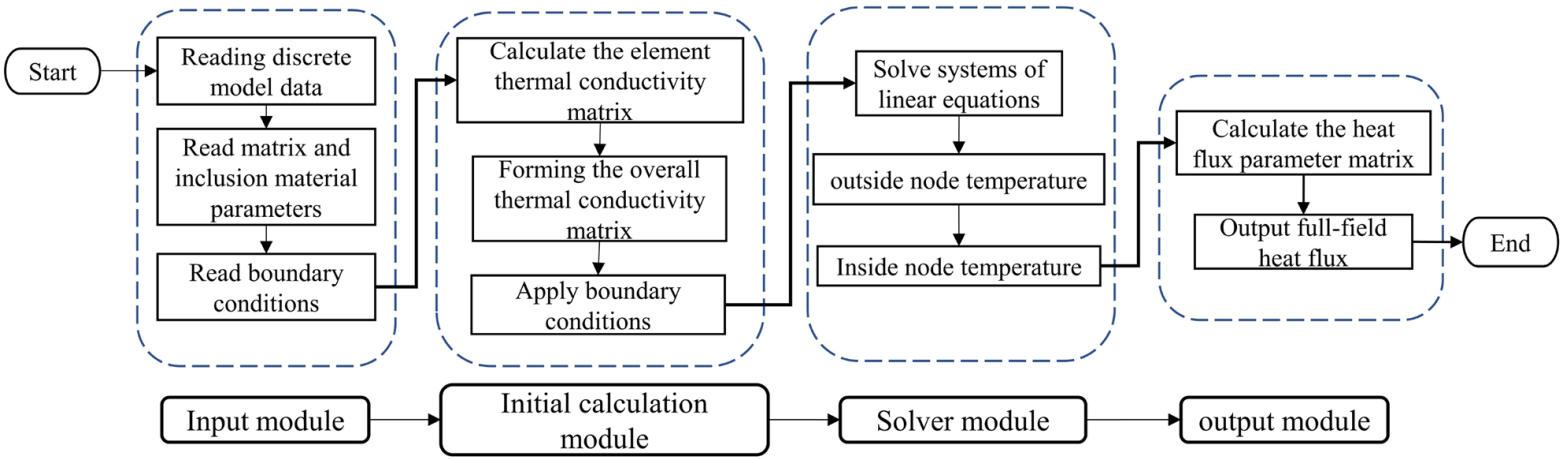
Flowchart of VCFEM calculation.

The computational structure of the program mainly consists of an input module, an initial computation module, a solution module and an output module. Its general calculation flow is shown in Fig. 3.

## Numerical examples

### A convergence study for heat flux

#### A convergence study of one element

A study is conducted to examine the sensitivity and convergence of the VCFEM. A square plate with side length of 100 mm containing an inclusion of radius 15 mm is studied. Equations (15) and (16) with various numbers of terms will be utilized for the computation to strike a balance between calculation efficiency and correctness. When the number of terms for Eqs. (15) and (16) is taken as 20 and 14, respectively, it is discovered that the VCFEM has greater computing efficiency with guaranteed correctness. Thus, the heat flux field in the inclusion is generated with a complete 6th order heat flux function Eq. (15) corresponding to 20 $\beta$ terms in the flux polynomial. The matrix flux field has an additional 14 reciprocal terms due to the 5^th^reciprocal heat flux function in Eq. (16). This result a total of 34 $\beta$ in matrix. The thermal conductivity k_m_ is 180 W/(mK), and the thermal conductivity k_c_ is 330 W/ (mK). Temperature of 10 K was applied to all nodes on the downside and temperature of 0 K was applied to all nodes on the upside.

**Figure 4:**
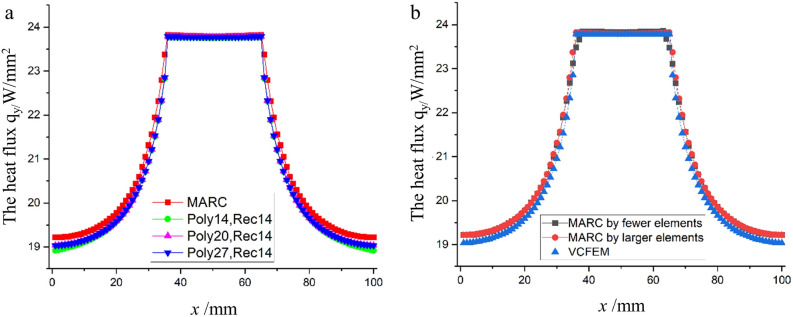
Flux distribution along *x*/L = 0.5. (**a**) Comparison of the results of different combinations of Poly and Rec terms with MARC calculations; (**b**) the results of VCFEM and MARC with different number of cells are calculated.

The results of the VCFEM calculations were compared with those of the finite element software MARC. For MARC, two groups of meshes are established, one is a coarse mesh with 3418 triangular elements, the other is a fine mesh with 7141 triangular elements. All the boundary conditions are the same of Voronoi mesh. A comparison of heat flux in y direction at mid-section) is shown in Fig. 4. It can be seen from the figure that the results calculated using a large number of 7141 finite elements are agreed well to those calculated by using VCFEM. However, the results calculated using fewer finite element of 3418 elements deviate from the accurate values. This proves high computational efficiency and accuracy of the proposed VCFEM.

**Figure 5:**
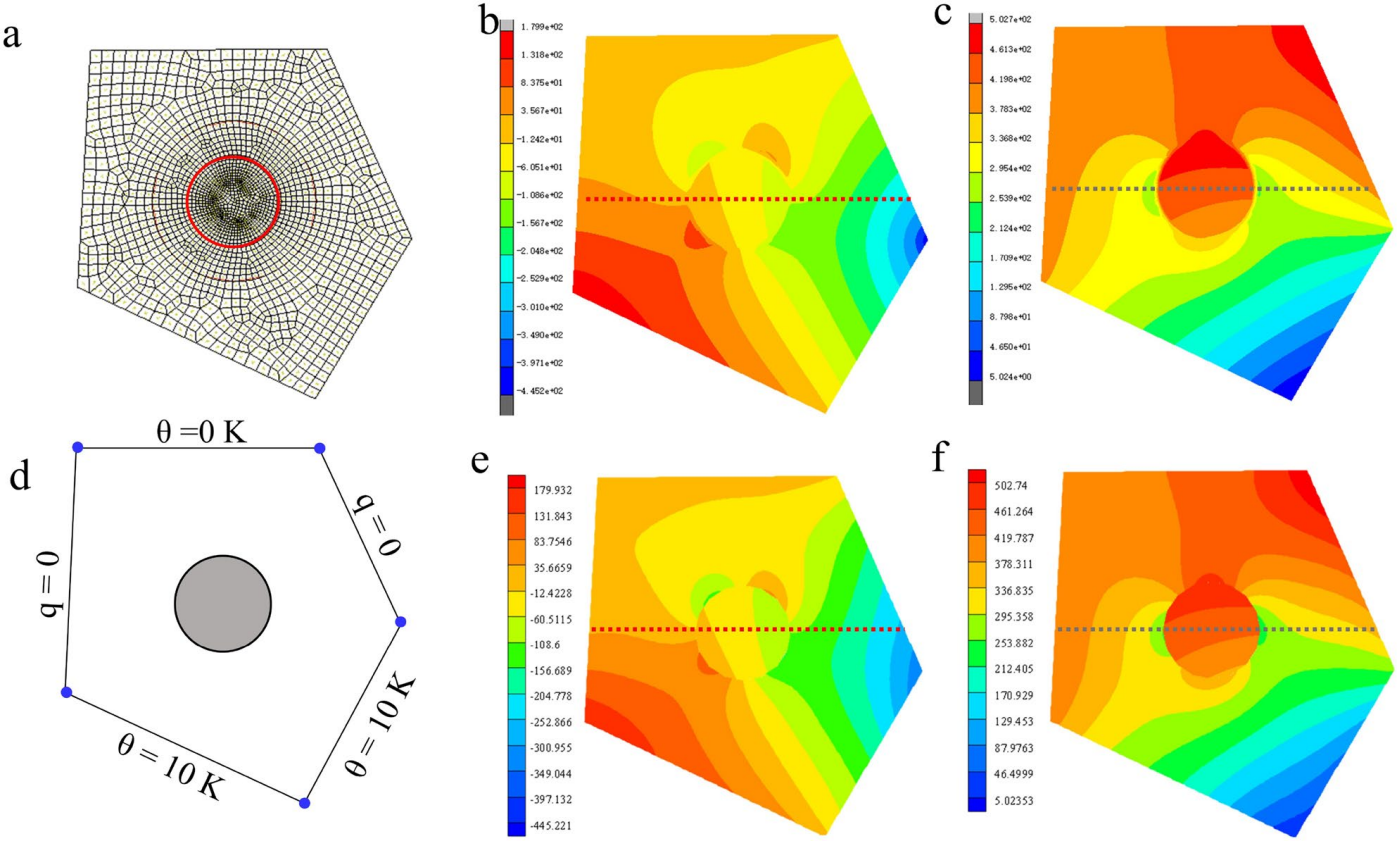
Mesh delineation and computational results for non-rectangular models. (**a,d**) MARC's meshing scheme with 4754 elements and a VCFEM cell; (**b,e**) horizontal heat flux q_x_ calculated by MARC and VCFEM; (**c,f**) vertical heat flux q_y_ calculated by MARC and VCFEM.

**Figure 6:**
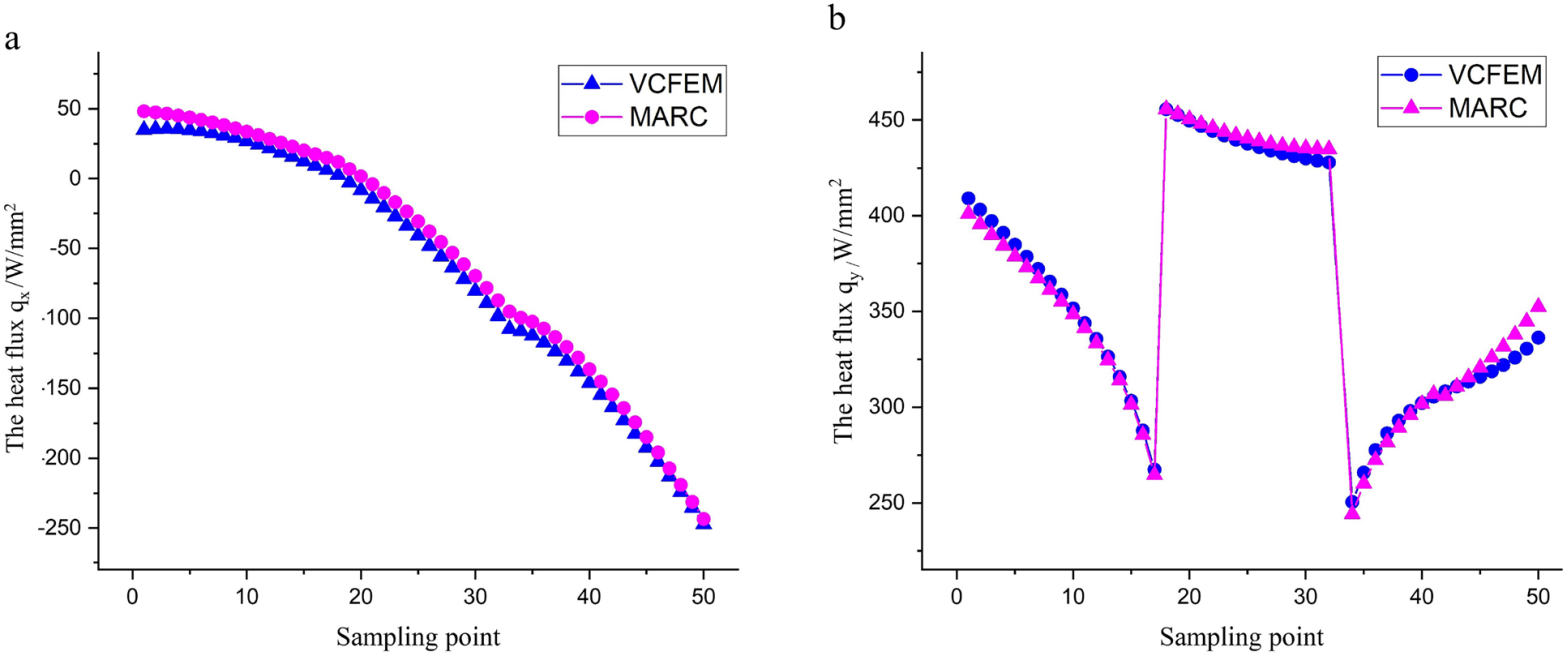
Heat flux on the sampling path in Fig. 5. (**a**) Horizontal heat flux q_x_ comparison results, (**b**) vertical heat flux q_y_ comparison results.

Furthermore, in order to verify the applicability of VCFEM to generally shaped materials, a non-rectangular shaped model was computed with VCFEM (Fig. 5d–f) and the results were compared with those of the finite element software MARC(Fig. 5a–c). Where 34β is still used for the matrix and 20 polynomial terms are used for inclusions. The cloud diagram of the heat flux is shown in Figs. 5, and 6 illustrates the comparison results on the sampling path in Fig. 5b. The results show that the VCFEM calculations are generally satisfactory for models with pointed/cornered profiles, but the accuracy at the pointed corners needs to be improved as shown at the right end of Fig. 6b.

#### Sensitivity to geometric distortion

**Figure 7:**
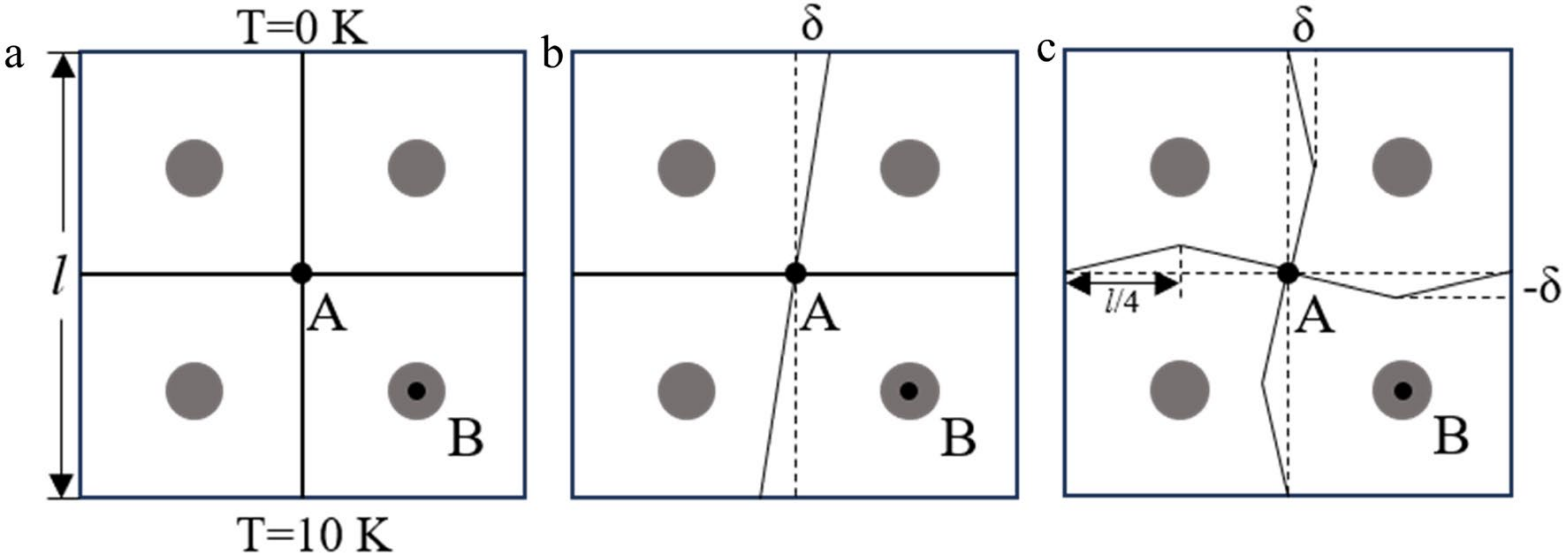
Meshing scheme for Voronoi elements: (**a**) normal Voronoi cells, (**b**) Voronoi cells with small linear geometric distortions and (**c**) Voronoi cells with small parabolic geometric distortions.

This section's primary goal is to examine, using the model with four Voronoi cells in Fig. 7, how sensitive Voronoi cells are to geometric distortions. The model's upper and lower boundaries have temperatures of 0 K and 10 K, respectively. The left and right boundaries are adiabatic, and the matrix and inclusions' thermal conductivities are 180 W/mK and 330 W/mK, respectively.

**Figure 8:**
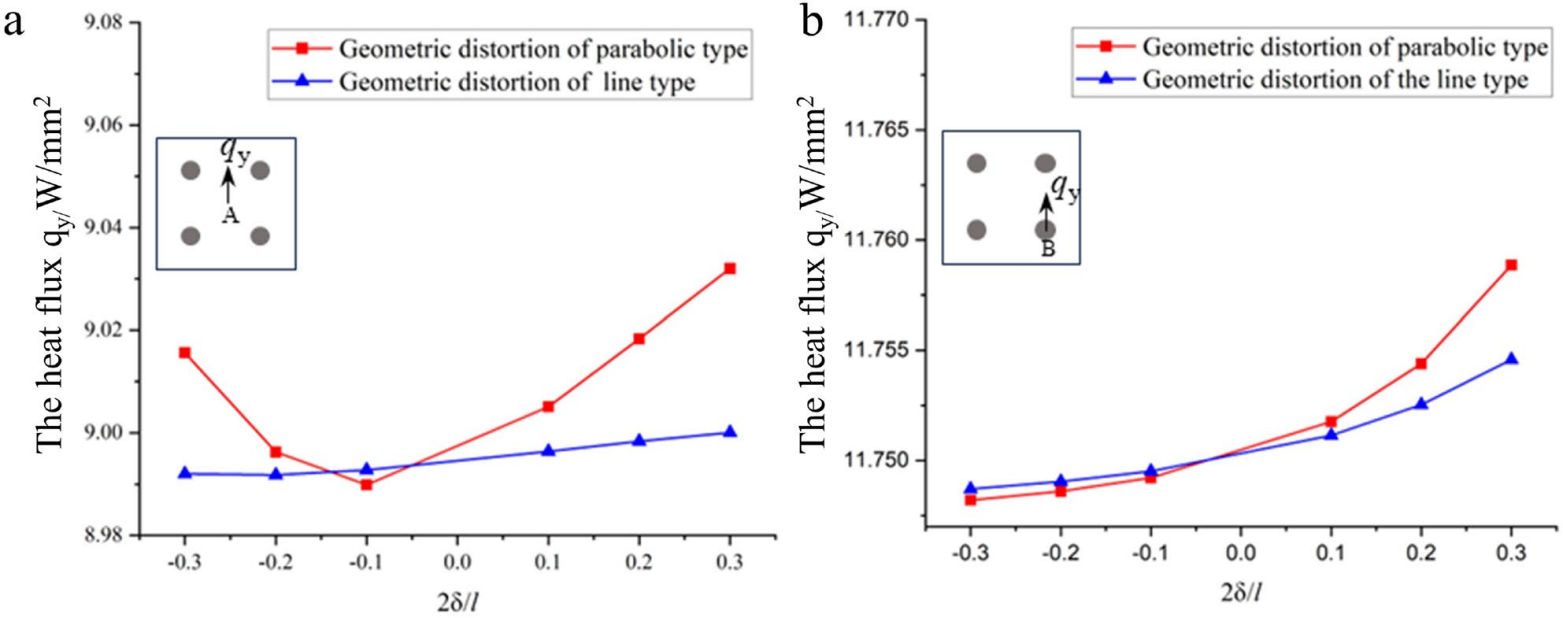
(**a**) Sensitivity of the heat flux density of the matrix at A to geometric distortion. (**b**) Sensitivity of the heat flux density of inclusions at B to geometric distortion.

Figure 7a shows the most ideal case, and the cells of Fig. 7b, c have been partitioned into the grid using linear and parabolic forms, respectively^14^. In the calculations, it is assumed that the values are positive when the resulting rightward and upward δ. The geometric distortion is determined by the parameter 2*δ*/*l*, which fluctuates in the range −0.3:0.3 to ignore as much as possible the effect due to the close distance between the inclusion and the cell boundary. Figure 7 shows how the heat flux varies with 2*δ*/*l* for the matrix and inclusions at A and B, respectively. Calculations in Fig. 8 demonstrate that when the mesh produces geometrical distortions, changes on the order of 10^–2^ are produced in the heat fluxes of the matrix and inclusions. As a result, the Voronoi cell's effect on geometrical distortion when calculating heat flux can be judged to be within an acceptable error range. This suggests that the effects of geometric distortion in conventional finite element methods have been somewhat mitigated by VCFEM.

#### A convergence study for RVE containing multiple inclusions

**Figure 9:**
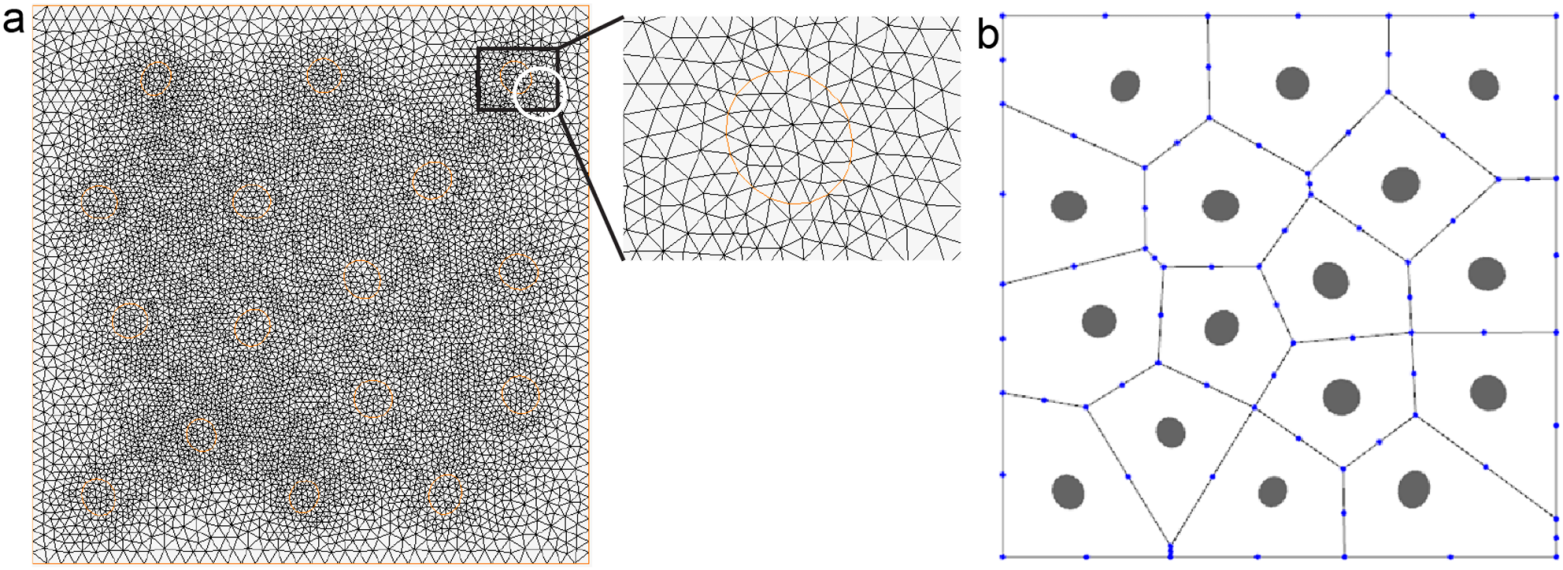
(**a**) Marc mesh and (**b**) Voronoi mesh with 16 inclusions.

**Figure 10:**
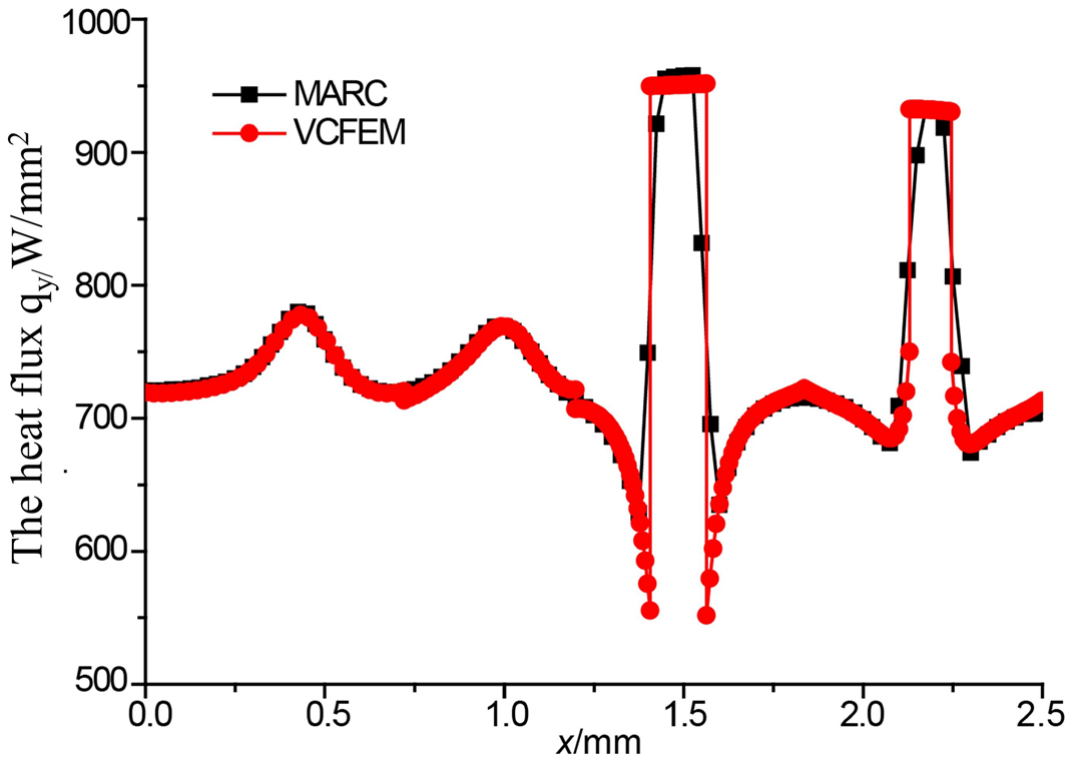
Flux distribution along y/L = 0.5.

**Figure 11:**
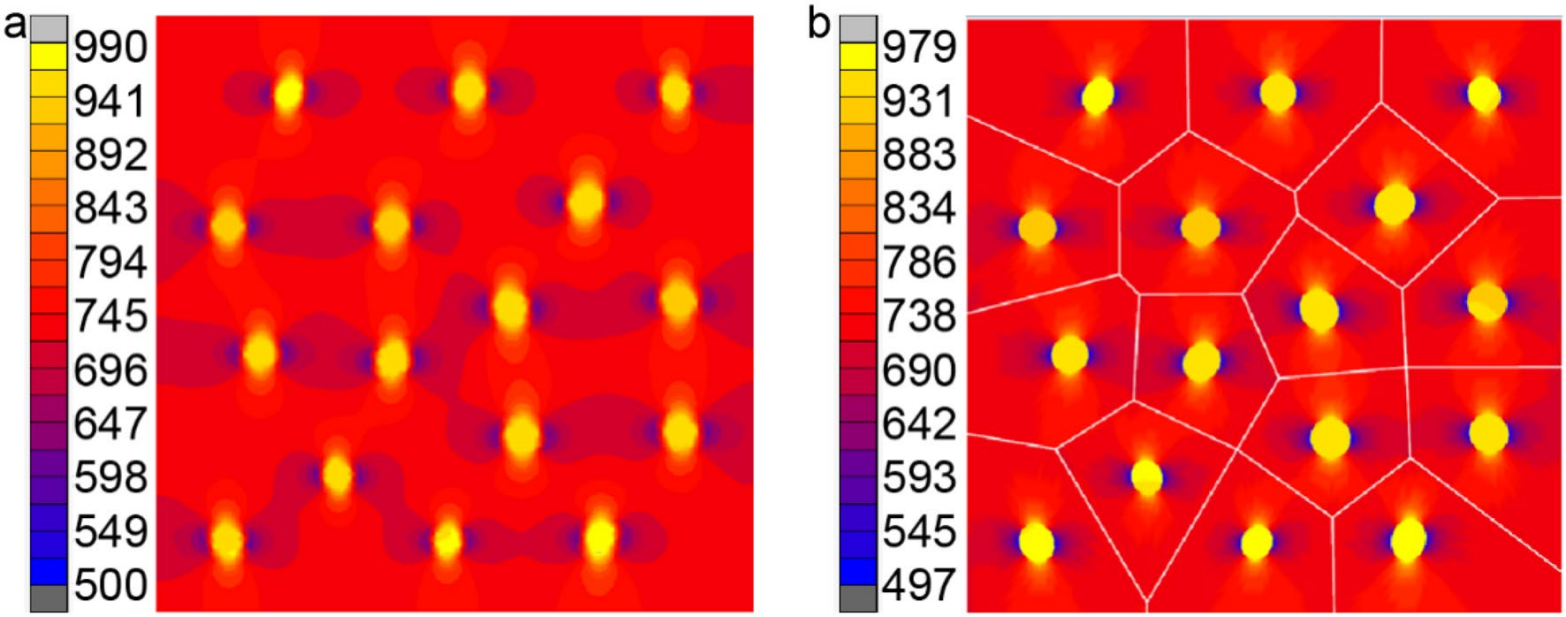
Contour plots of the flux q_y_ with (**a**) MARC and (**b**) VCFEM.

In this example, RVE (2.5 mm × 2.5 mm) containing 16 elliptical inclusions with size and location randomly distributed is considered. The volume fraction of inclusions is 5%. Figure 9 shows mesh by MARC and VCFEM models. The MARC mesh consists of 14,860 Tri elements while the VCFEM mesh has only 16 elements corresponding to the number of inclusions. The thermal conductivity of the matrix is 180W/mK and the thermal conductivity of inclusions is 330W/mK. A temperature of 10 K is applied to the bottom edge and a temperature of 0 K is applied to the top edge of the model. Figure 10 shows the true microscopic heat flux at the mid-section(y/L = 0.5). Full field heat flux distribution is shown in Fig. 11.

### Effect of microstructure on response

In this section the effect of volume fraction, shape, thermal conductivity and number of inclusions, on the macroscopic as well as true microstructural response of RVEs is studied. Representative volume elements (RVE) characterize a point by microstructure. The effective heat conductive of RVE correspond to average heat flux divided by the temperature gradient. The macroscopic average heat flux corresponds to the volume average of the microscopic heat flux. When apply fixed temperature $\Theta_{2}$ on the upside and $\Theta_{1}$ on the downside, the vertical average heat flux of the RVE is calculated as

33 $$\overline{q}_{y} = \frac{{\int_{RME} {q_{y} d\Omega } }}{{\int_{RME} {d\Omega } }} = \frac{{\sum {\iint {q_{y} (x,y)dxdy}} }}{{{\text{L}}^{2} }}$$

where, L is the side length of a square RVM.

The temperature gradient in the Y direction was calculated from:

34 $$\overline{\varepsilon }_{y} = (\Theta_{1} - \Theta_{2} )/L$$

The Voronoi cell finite element model uses 34 $\beta$ parameters in the heat flux interpolation for matrix and 20 $\beta$ parameters in the heat flux interpolation for inclusion for all case parameters in the stress interpolation for all cases.

#### Effect of volume fraction of inclusion

**Figure 12:**
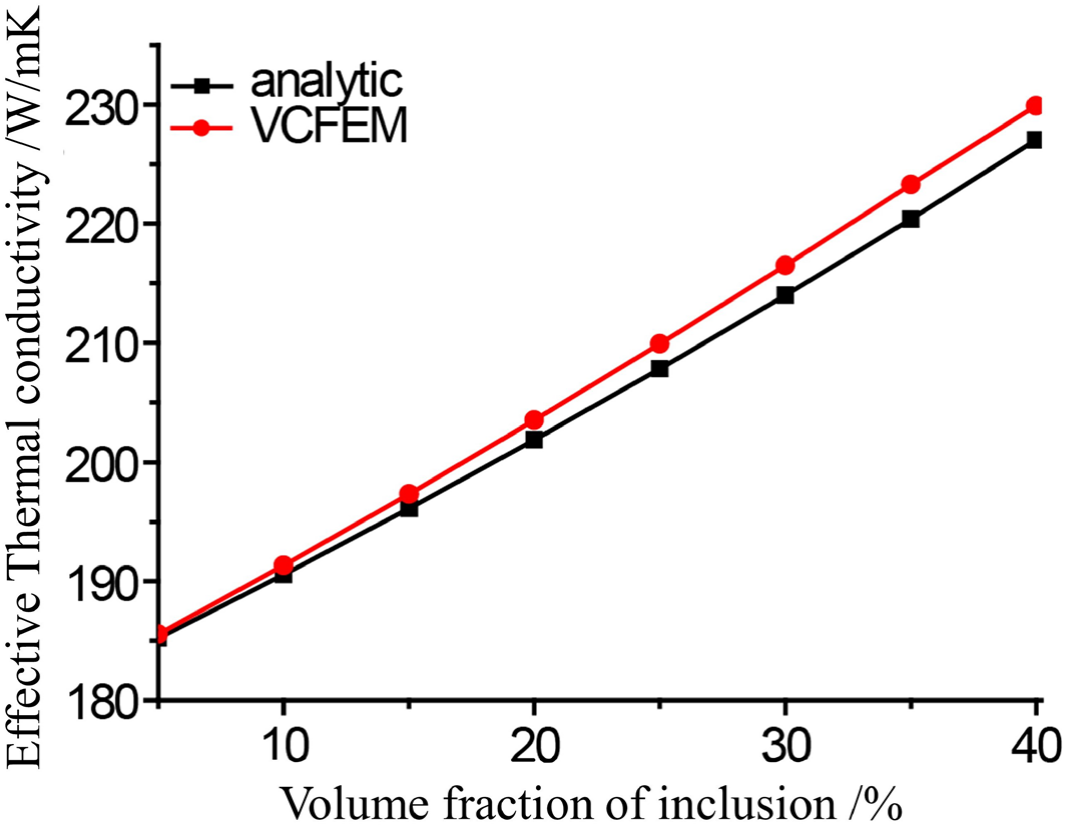
k_e_ computed by VCFEM and analytical method.

**Figure 13:**
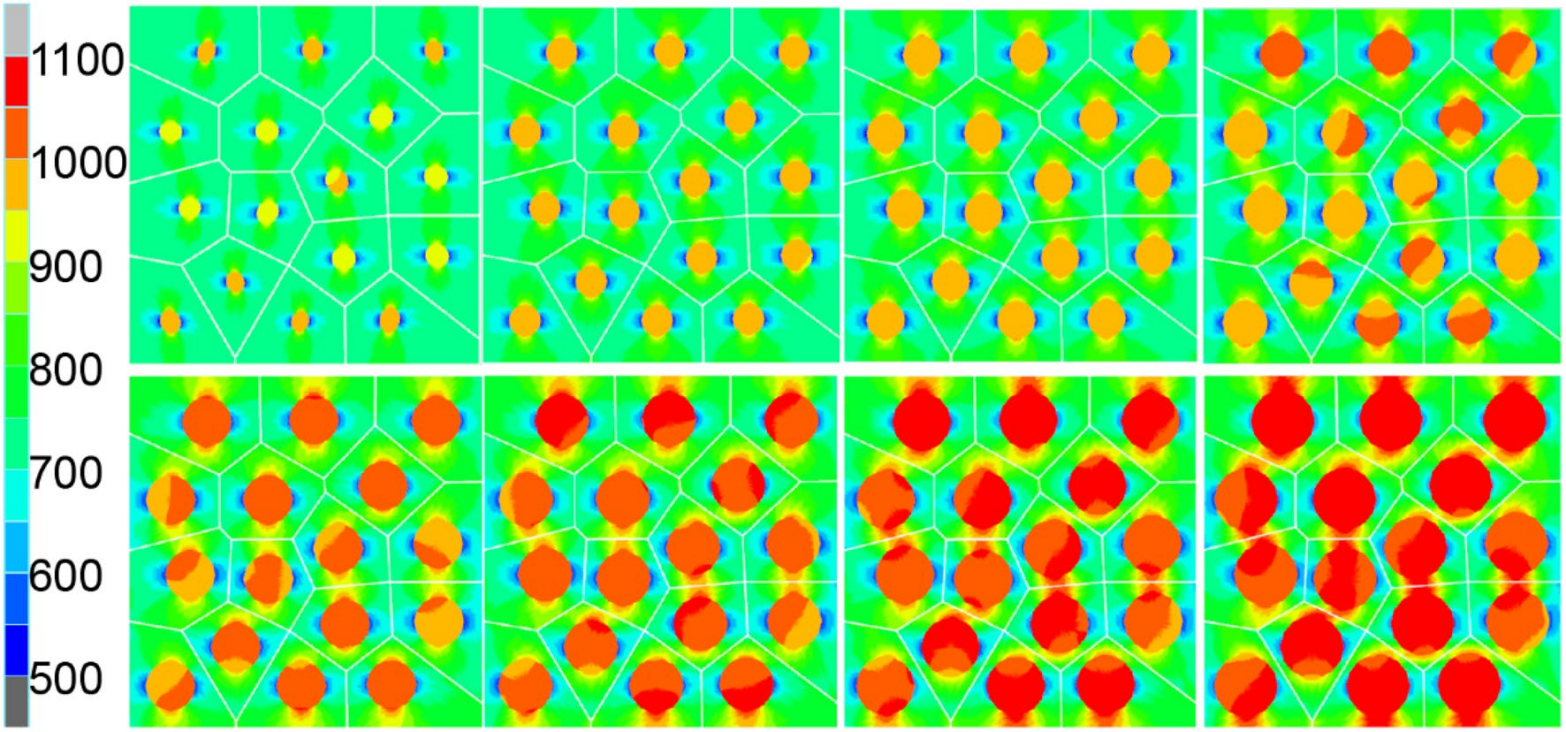
Heat flux fields in y direction with volume fracture of inclusion varies from 5 to 40% at 5% intervals.

In this study, the thermal conductivity k_m_ of matrix is 180 W/mK, and the thermal conductivity k_*c*_ of reinforcement is 330 m W/mK. Various volume fractions of circular inclusions are considered. The macroscopic response effective thermal conductivity $({{\text{k}}}_{e})$ increases with the increase of inclusion volume fraction, as shown in the Fig. 12. This is expected since larger volume of the inclusions will cause the overall response to move in the direction of its individual response. At the same time, the analytical value calculated by flexible model^12^ as Eq. (35) is also drawn in the Fig. 12. When the volume fraction of inclusion is 5%, the relative error between the value calculated by VCFEM and the analytical value is 0.1%, when the volume fraction of inclusion is 40%, the relative error between the value calculated by VCFEM and the analytical value is 1.4%. Figure 13 shows the y-direction heat flux cloud map corresponding to the volume fraction of the inclusions mentioned above.

35 $$k_{e} = \frac{1}{f - 2}\left\{ {A + \sqrt {A^{2} + 2\left( {f - 2} \right)k_{m} k_{c} } } \right\}$$

where,$A = \left( {\frac{f}{2}v_{m} - 1} \right)k_{m} + \left( {\frac{f}{2}v_{c} - 1} \right)k_{c}$, *v*_m_ and *v*_c_ are the volume fractions of matrix and inclusions, respectively, and the flexible factor^12^ f = 4.5.

#### Effect of shape, orientation and thermal conductivity of inclusion

In this study, the thermal conductivity k_*m*_ of matrix is 180 W/(m K), and the thermal conductivity k_*c*_ of reinforcement varies from 30 to 480 W/(m K) and increases gradually at intervals of 50 W/(mK). The effect of shape and orientation together with of thermal conductivity of inclusions on the behavior of microstructure and macrostructure is investigated respectively. Sixteen elliptical inclusions of various shapes, orientation and thermal conductivity but with a constant volume fraction of inclusion of 5%, are distributed randomly in the RVE. Figure 9b shows the VCFEM mesh for the fifth group, and Fig. 15 shows the VCFEM meshes for the other four different distributions. They are

1. major axis in the horizontal direction
2. major axis in the vertical direction
3. major axis rotates 45° from the horizontal direction
4. major axis rotates 145° from the horizontal direction
5. arbitrary shapes, sizes and orientation.

The ratio of major to the minor axis, for ellipses in the last four cases is 2.3. For each of the five groups, the effective thermal conductivity changing with different thermal conductivity of particles were calculated and shown in Fig. 2. It can be seen from the Fig. 2 that when the thermal conductivity of the particles increases from 30W/mK to 270 W/mK, k_*e*_ of the composite material shows an increasing trend. however, the effects of particle shape on the effective thermal conductivity are different with different particle thermal conductivity.

**Figure 14:**
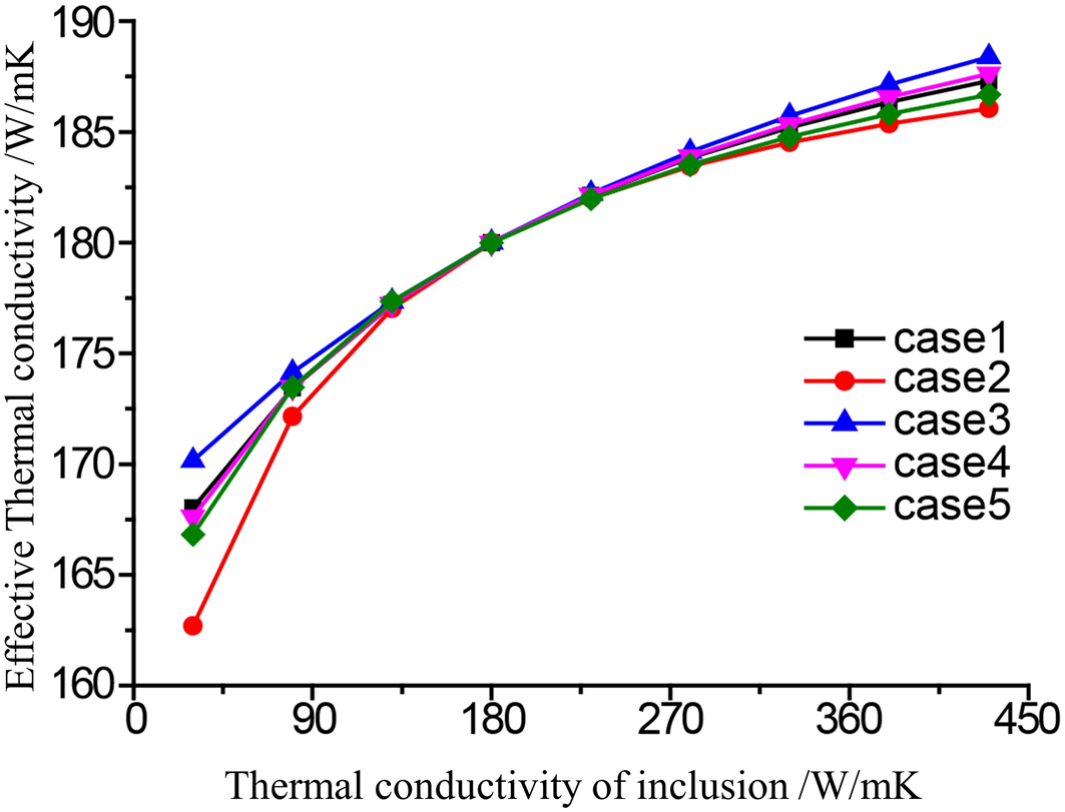
k_e_ varies with different inclusion shape and Kc.

**Figure 15:**
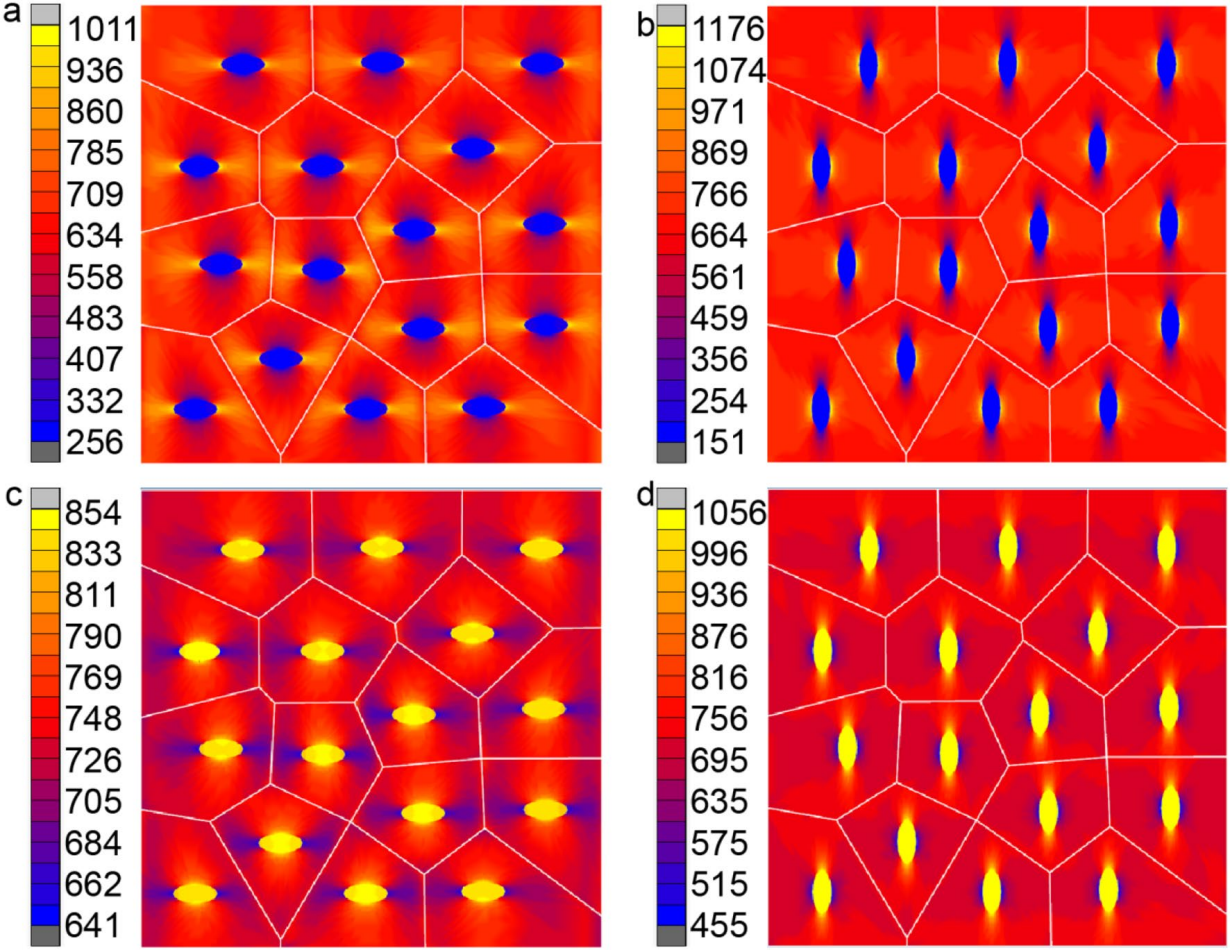
Heat flux in y direction with k_m_ = 180(W/mK) and k_c_ = 330(W/mK) for (**a**) group 1, (**b**) group 2, (**c**) group 3 and (**d**) group 4.

1. When 30W/mK ≤ k_*c*_ < 130 W/mK, the shape and direction of the particles have great influence on the macroscopic k_*e*_ as shown in Fig. 14. At this interval, the k_m_ is higher, the K_*c*_ is lower, and the matrix is the main carrier for conducting heat. When the orientation of the particles is different and the distance between the edges of the particle’s changes, the width and path of the heat conduction channel of the matrix will change. When the particles are distributed in the group 1, the distance between the edges of the particles is small, thus the width of the heat conduction channel of the matrix becomes narrow, and the matrix is hindered more on the heat conduction path. Therefore, k_*e*_ of the composite material is reduced, and k_*e*_ of microstructures of the group 2 is 162.69 W/mK which is the smallest among the five groups, and k_*e*_ of the group 3 is 170.16 W/mK which is the largest. The difference between the maximum value and the minimum value is 7.47W/mK. k_*e*_ of other microstructures is between therm. Taking k_*m*_ = 180 W/mK, *k*_*c*_ = 30 W/mK for example, Fig. 15 is a contour of microscopic heat flux distribution of composite materials for group 1 and group 2 simulated by VCFEM. From Fig. 15 we can see that when the heat flux meets the particles with low thermal conductivity, only a small part of the heat flux enters into the particles, and the heat flux density inside the particles is the lowest. Some of the heat flux bypasses the particles, and the heat flux density reaches the maximum at the position to the right and left edges of particles. The difference between the peak values of group 1 and 2 is 165 W/mK.
2. When 130 ≤ *K*_*c*_ ≤ 230, that is k_c_ is similar to k_*m*_, k_e_ of the composite seems not changes with various microstructures by changing the inclusion orientation.
3. When 230W/mK ≤ k_*c*_ < 480 W/mK, the shape and direction of the particles have great influence on the *k*_*e*_ as shown in Fig. 14. At this interval, k_*c*_ is higher than k_*m*_. The matrix and inclusion are both the main carrier for conducting heat. k_*e*_ of the composite material is increased. Effect of inclusion orientation on k_*e*_ is almost the same as that of lower k_*c*._ k_*e*_ of microstructures of the group 1 is 186.61 W/mK which is the smallest among the five groups, and k_*e*_ of the group 2 is 189.48 W/mK which is the largest. The difference between the maximum value and the minimum value is 2.87 W/mK. Taking k_m_ = 180 W/mK, k_c_ = 330 W/mK for example, Fig. 15a, b is a contour of microscopic heat flux distribution of composite materials for group 1 and group 2 simulated by VCFEM. From Fig. 15 we can see that when the heat flux meets the particles with high thermal conductivity, a great of the heat flux enters into the particles, and the heat flux density inside the particles is the highest. Some of the heat flux bypasses the particles, and the heat flux density reaches the minimum at the position to the right and left edges of particles.

It can therefore be conducted that microscopic as well as macroscopic response is highly sensitive to the distribution of shapes of inclusions when *k*_*c*_ is deviated from k_*m*_, while those response is insensitive to the distribution of shapes of inclusions when *k*_*c*_ is equivalent with k_*m*._

#### Effect of inclusion numbers

**Figure 16:**
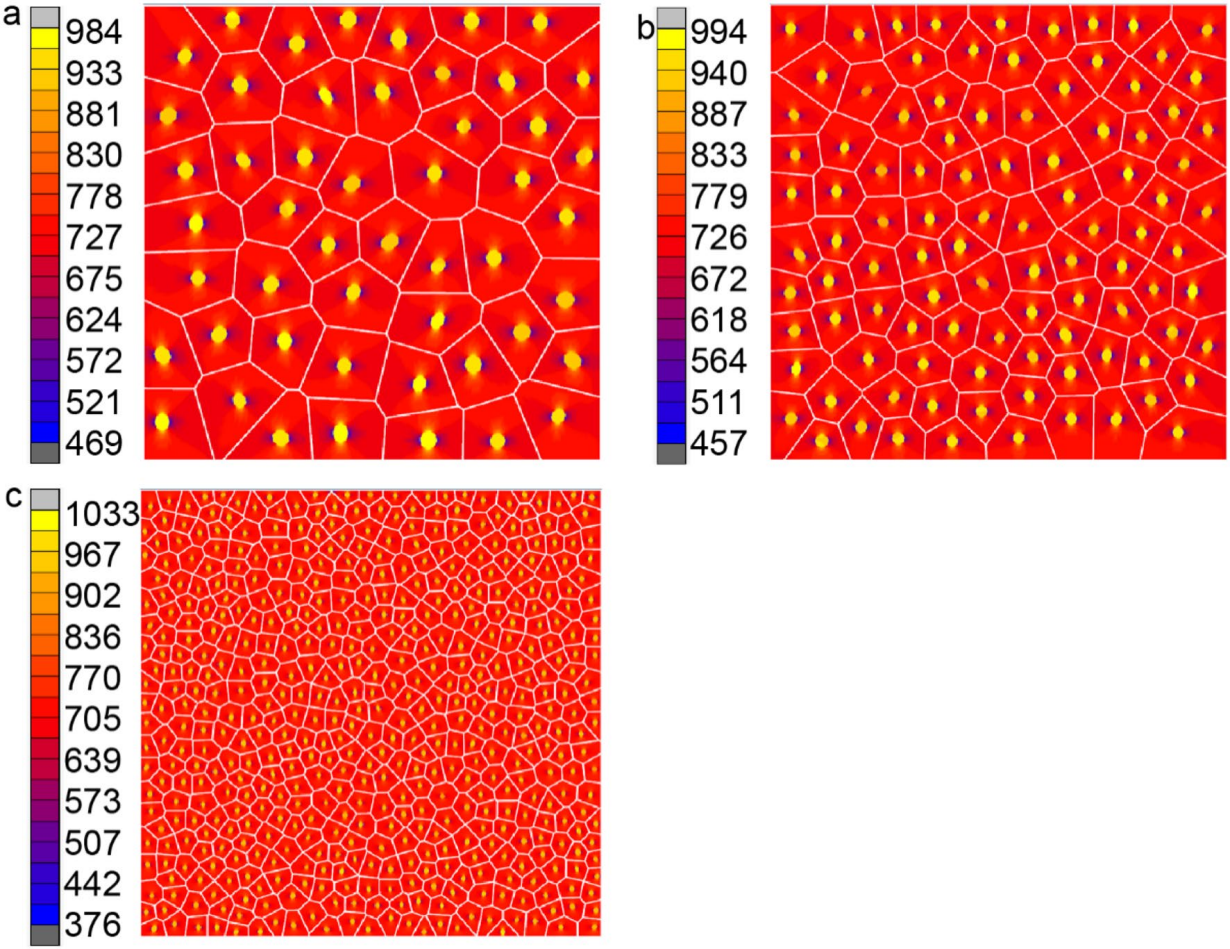
Heat flux in y direction with k_m_ = 180(W/mK) and k_c_ = 330(W/mK) for (**a**) 50 inclusions, (**b**) 100 inclusions and (**c**) 500 inclusions.

In this study, the effect of number of inclusions on the behavior of microstructure and macrostructure is investigated. Inclusions of various shapes, sizes and orientation are distributed randomly in the RVE. Four groups of different inclusion numbers with a constant volume fraction of ***V***_***c***_** = **5%, are studied. These four groups contain50, 100, 500 and 16 inclusions respectively. Matrix k_*m*_ = 180 W/mK and inclusion k_*c*_ = 330 W/mK are used for this study. Contour plot of microstructures with different number of 50, 100 and 500 inclusions are shown in Fig. 16, while 16 inclusions is shown in Fig. 16b. From these figures, it can be seen that the heat flux distribution of microstructures containing 16, 50 and 100 inclusions is very similar, the difference between the maximum value is very small, and the difference between the minimum values is also very small. There is gap between the minimum heat flux for the RVE containing 500 inclusions and others. Macroscopic effective thermal conductivity for those four RVEs are listed in Table 1. From these data, it can be seen that k_*e*_ is insensitive to the number of inclusions.

**Table 1:** Effective thermal conductivity k_e_ computed by VCFEM for various number of inclusions.

Number of inclusions	16 inclusions	50 inclusions	100 inclusions	500 inclusions
k_e_ (W/mK)	185.25	185.202	185.186	185.195

## Conclusions

This paper develops a VCFEM based on hybrid flux model and direct constraint method for steady-state heat conduction problem of composites imbedded with inclusions. The VCFEM is based on a two-field parameter function, that makes independent assumptions on the heat flux and boundary temperature fields in each element. Heat flux continuous on matrix-inclusion interface and inter-element boundary are relaxed and directly introduced in the functional by temperature as Lagrange. The accuracy and efficiency of VCFEM are verified by comparison with conventional finite element method inserted in MARC. In addition, the study of cells that produce geometric distortions in both linear and parabolic forms exemplifies the fact that geometric distortions will have a smaller effect in VCFEM.

Influence of microstructure morphology such as inclusion shape, size, and distribution on heat flux distribution and effective heat conductivity are studied by VCFEM.

It is observed that the ${{\text{k}}}_{{\text{e}}}$ of the composite tends to inclusion direction with the increase of volume percentage of inclusions. The shape and orientation of inclusions have great influence on the ${{\text{k}}}_{e}$ of composite materials, when the properties of inclusions are different from matrix, which is usually exist in real microstructures. When the volume fraction of inclusion is constant, the number of inclusions has little effect on the ${{\text{k}}}_{{\text{e}}}$ of composite materials, however, it has great influence on microstructure. In conclusion, VCFEM is very effective for analyzing composite materials with randomly distributed inclusion which often occur in actual material.

## Data Availability

The data that support the findings of this study are available from the first author and the corresponding author, upon reasonable request.
